# Supplementary material for: A Streamlined Approach for Fluorescence Labelling of Low-Copy-Number Plasmids for Determination of Conjugation Frequency by Flow Cytometry
Source: Microorganisms. 2023 Mar 29;11(4):878. doi: 10.3390/microorganisms11040878 (PMC10144549; doi:10.3390/microorganisms11040878)
Supplement: Supplementary file 1 [file microorganisms-11-00878-s001.zip › microorganisms-2240613-supplementary.pdf]

Supplementary Materials

A Streamlined Approach for Fluorescence Labelling of Low-Copy-Number Plasmids for Determination of Conjugation Frequency by Flow Cytometry

Qin Qi [1,†], Muhammad Kamruzzaman [1,*] and Jonathan R. Iredell [1,2,*]

1 Centre for Infectious Diseases and Microbiology, The Westmead Institute for Medical Research, The University of Sydney, Westmead, Sydney, NSW 2145, Australia; qin.qi@sydney.edu.au
2 Westmead Hospital, Westmead, Sydney, NSW 2145, Australia
* Correspondence: muhammad.kamruzzaman@sydney.edu.au (M.K.); jonathan.iredell@sydney.edu.au (J.R.I.)
† Current address: School of Natural Sciences, Macquarie University, Sydney, NWS 2109, Australia.

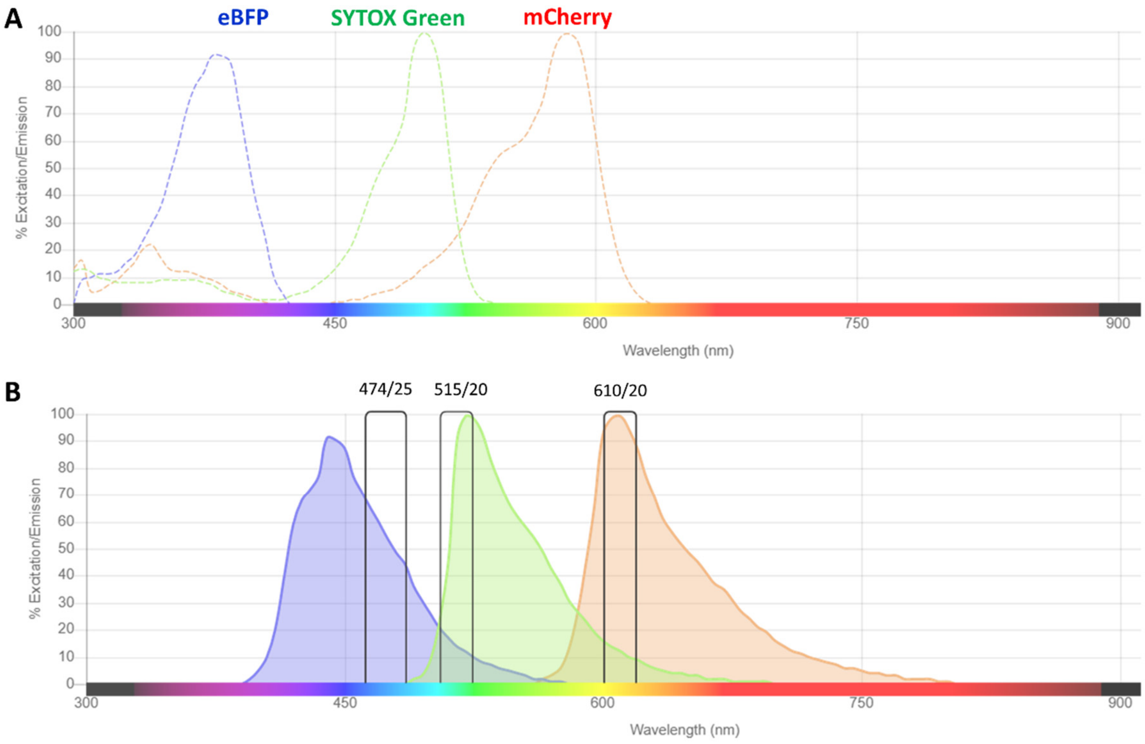

Figure S1. Fluorescence excitation-emission spectra of three fluorophores. (**A**) The excitation spectra for eBFP, SYTOX Green and mCherry are represented by the blue, green and red lines respectively on the Fluorescence Spectrum Analyzer for Flow Cytometry plot (Beckman Coulter Life Sciences, Indianapolis, IN, USA). (**B**) The emission spectra for eBFP, SYTOX Green and mCherry are shaded in blue, green and red respectively. The positions of the wavelength ranges of bandpass filters used in FACSymphony flow cytometer for mCherry (610 ± 20 nm) and BFP detection (474 ± 25 nm), as well as the that used in CytoFLEX for SYTOX Green detection (515 ± 20 nm) are shown by the rectangles.

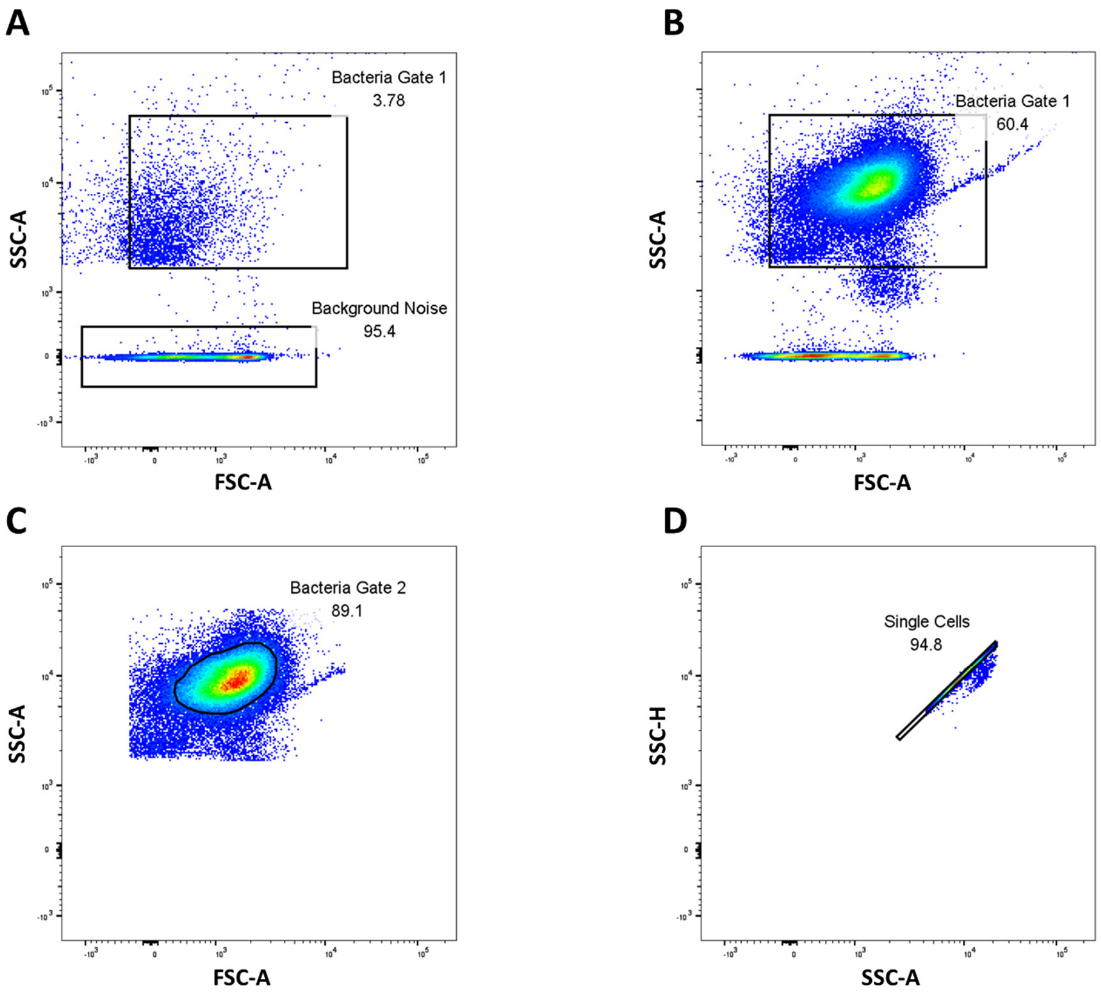

Figure S2. Gating strategy for flow cytometry. (**A**,**B**): A comparison between the FSC-H vs. SSC-H bivariate plots for sterile-filtered PBS + 0.2 mM EDTA blank medium control (Panel A) and an *E. coli* bacteria-containing sample (the UB5201Rf + pConj_blue_strong strain is shown in Panels B-D as an example) showed that background noise events in the PBS medium and bacterial cell events formed two distinct clusters. A universal rectangular gate "Bacteria Gate 1" was drawn and applied to all samples. **(C)** The autogating tool in the FlowJo analysis software was used to capture approximately 90% of all bacterial cell events based on the contours of each cluster within "Bacteria Gate 1" in every bacteria-containing sample. This required separate "Bacteria Gate 2" contour-shaped gates to be determined for each individual sample. Compensation matrices were then calculated using the non-fluorescent J53Az negative control strain and the single-color positive control strains. **(D)** Doublet discrimination was performed on SSC-H vs. SSC-A bivariate plots for drawing a narrow gate along the diagonal of the plot to exclude events that deviated from the linear relationship between SSC-H and SSC-A. A universal spider gate was drawn on red (mCherry) versus blue (BFP) fluorescence bivariate plots in a way that clearly allowed the single-color positive control strains to be distinguished from the non-fluorescent J53Az negative control strain as shown in Figures 1 and 3. The percentage of false positive events in each region (Q1–Q4) should generally be kept below 2%.
